# Supplementary material for: The Impact of Prolonged Inflammation on Wound Healing
Source: Biomedicines. 2022 Apr 6;10(4):856. doi: 10.3390/biomedicines10040856 (PMC9025535; doi:10.3390/biomedicines10040856)
Supplement: Supplementary file 1 [file biomedicines-10-00856-s001.zip › biomedicines-1642626-supplementary.pdf]

Supplementary to the Manuscript „The impact of prolonged inflammation on wound healing", Holzer-Geissler et al.

Composition and parameters of the wound scores used for the present study.

Inflammation score

Supplementary Table S1 – Inflammation Score. The inflammation score evaluates the presence of pus, erythema and swelling, as well as the width of the erythema giving extra points. The scoring of erythema is adapted from the scoring scheme of Halin et al (American Journal of Pathology 2008, (1)) and the inflammation score adapted from the wound score of Matsui et al (Wound Repair and Regeneration 2011, (2)).

**Parameter**	**Phenotype**	**Score**
Pus	None	0
	Present	1
Erythema	None	0
	Mild	1
	Moderate	2
	Pronounced	3
	Severe	4
Erythema Width	<5mm	0
	≥5mm	1
Swelling	None	0
	Mild	1
	Severe	2

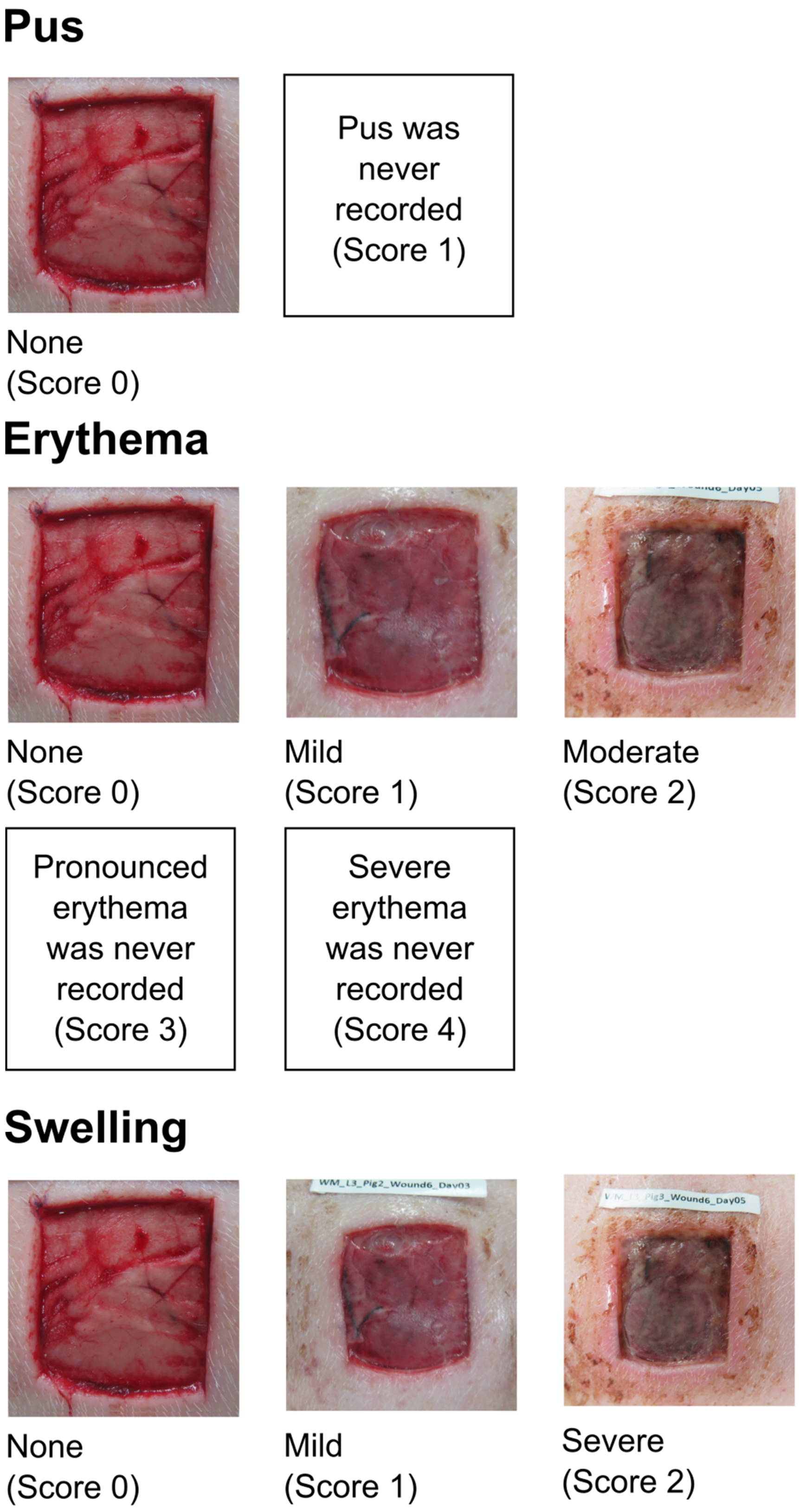

Supplementary Figure S1 – Inflammation Score. Representative pictures of the wounds to exemplify the scoring scheme and points given of the inflammation score.

Supplementary Table S2 – Area Score. The area score evaluates the state of the wound, rating it as dry or moist, and the size. The presence and amount of granulation tissue as well as necrosis is scored. The area score is adapted from the wound score of Matsui et al (Wound Repair and Regeneration 2011, (2)).

Parameter	Phenotype	Score
Wound	Dry	0
	Moist	1
Size	≤25 mm	0
	≤30 mm	1
	≤35 mm	2
	>35 mm	3
Granulation tissue	Covering wound	0
	Moderate	1
	None	2
Scab	Fallen off	0
	Moderate	1
	Mild	2
	None	3
Necrosis	None	0
	Mild	1
	Moderate	2
	Pronounced	3
	Severe	4

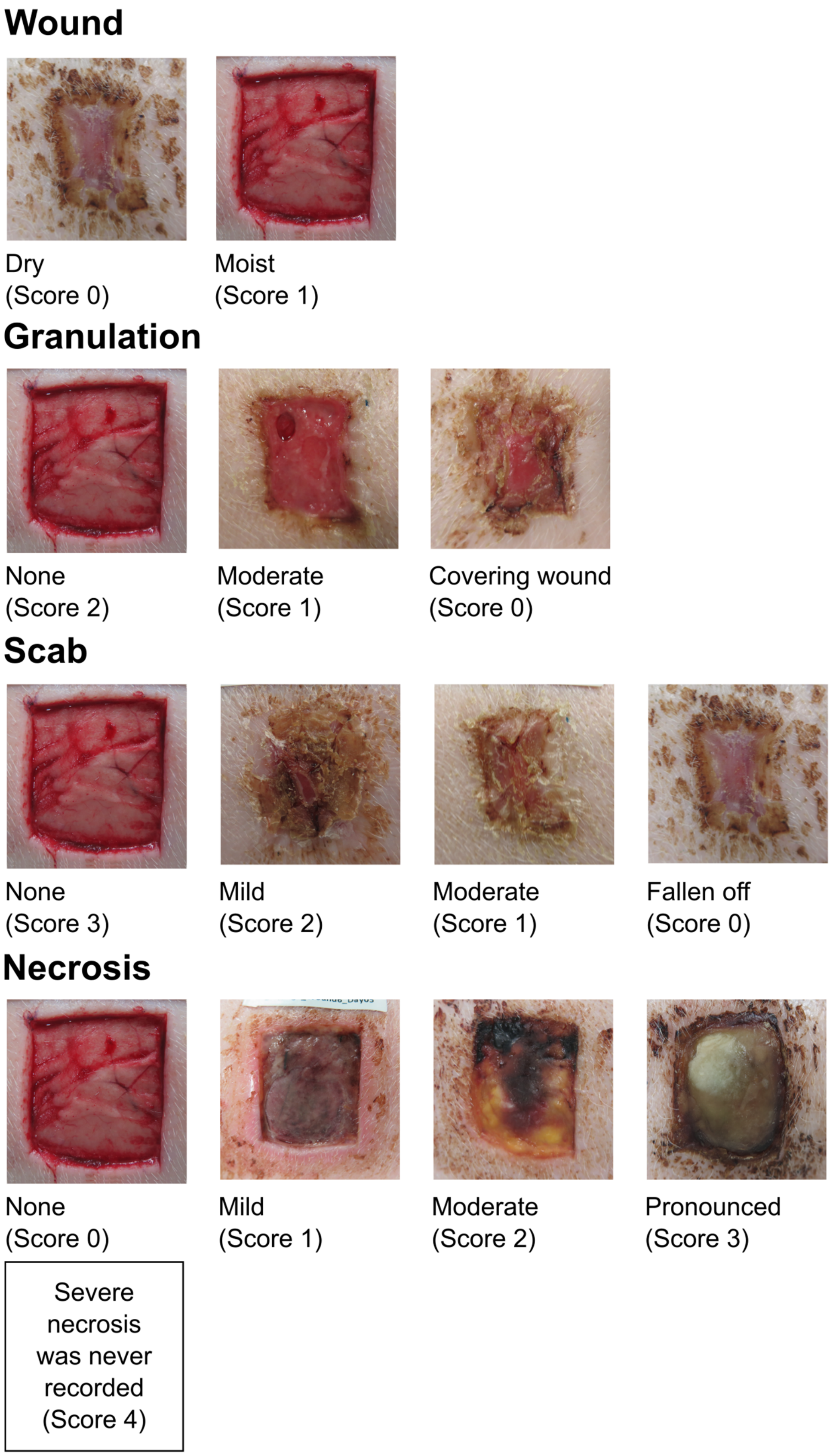

Supplementary Figure S2 – Area Score. Representative pictures of the wounds to exemplify the scoring scheme and points given of the area score.
